# Non-destructive monitoring of maize LAI by fusing UAV spectral and textural features

**DOI:** 10.3389/fpls.2023.1158837

**Published:** 2023-03-31

**Authors:** Xinkai Sun, Zhongyu Yang, Pengyan Su, Kunxi Wei, Zhigang Wang, Chenbo Yang, Chao Wang, Mingxing Qin, Lujie Xiao, Wude Yang, Meijun Zhang, Xiaoyan Song, Meichen Feng

**Affiliations:** ^1^ College of Agriculture, Shanxi Agricultural University, Jinzhong, Taigu, Shanxi, China; ^2^ College of Resources and Environment, Shanxi Agricultural University, Jinzhong, Taigu, Shanxi, China

**Keywords:** UAV, multispectral images, leaf area index, spectral feature, texture feature, maize

## Abstract

Leaf area index (LAI) is an essential indicator for crop growth monitoring and yield prediction. Real-time, non-destructive, and accurate monitoring of crop LAI is of great significance for intelligent decision-making on crop fertilization, irrigation, as well as for predicting and warning grain productivity. This study aims to investigate the feasibility of using spectral and texture features from unmanned aerial vehicle (UAV) multispectral imagery combined with machine learning modeling methods to achieve maize LAI estimation. In this study, remote sensing monitoring of maize LAI was carried out based on a UAV high-throughput phenotyping platform using different varieties of maize as the research target. Firstly, the spectral parameters and texture features were extracted from the UAV multispectral images, and the Normalized Difference Texture Index (NDTI), Difference Texture Index (DTI) and Ratio Texture Index (RTI) were constructed by linear calculation of texture features. Then, the correlation between LAI and spectral parameters, texture features and texture indices were analyzed, and the image features with strong correlation were screened out. Finally, combined with machine learning method, LAI estimation models of different types of input variables were constructed, and the effect of image features combination on LAI estimation was evaluated. The results revealed that the vegetation indices based on the red (650 nm), red-edge (705 nm) and NIR (842 nm) bands had high correlation coefficients with LAI. The correlation between the linearly transformed texture features and LAI was significantly improved. Besides, machine learning models combining spectral and texture features have the best performance. Support Vector Machine (SVM) models of vegetation and texture indices are the best in terms of fit, stability and estimation accuracy (R^2^ = 0.813, RMSE = 0.297, RPD = 2.084). The results of this study were conducive to improving the efficiency of maize variety selection and provide some reference for UAV high-throughput phenotyping technology for fine crop management at the field plot scale. The results give evidence of the breeding efficiency of maize varieties and provide a certain reference for UAV high-throughput phenotypic technology in crop management at the field scale.

## 1 Introduction

Maize is one of the essential food crops in the world, and its production mode and planting area are related to world food security (Tester and Langridge, 2010). Leaf area index (LAI) refers to the ratio of total green leaf area per unit land area to the unit land area (Ke et al., 2016), which is one of the important indexes for evaluating crop growth and ecological environment research (Richardson et al., 2011). It is not only closely related to crop photosynthesis (Duncan, 1971) and transpiration (Kang et al., 2003), but also often used as one of the basis for yield estimation (Duan et al., 2019; Fu et al., 2020). Therefore, efficient and precise monitoring of maize LAI is crucial for gaining insights into maize growth dynamics and optimizing maize breeding strategies.

However, the traditional methods to obtain LAI are mostly destructive sampling methods, using leaf area meters to measure isolated blades and calculate them (Yang et al., 2021). This method is time-consuming, laborious and inefficient. Although non-destructive monitoring of LAI can be achieved using handheld instruments such as the SunScan Canopy Analyser (Oguntunde et al., 2012) and the LAI-2200 (Wilhelm et al., 2000), the data obtained using handheld instruments only represent LAI at a small scale, making it difficult to realize rapid and nondestructive monitoring at field scale (Liu et al., 2016). Satellite remote sensing enables rapid and non-destructive monitoring of crop LAI at a regional scale. (Chen et al., 2010). However, its susceptibility to adverse weather conditions, low temporal and spatial resolution limits its ability to meet the quantitative monitoring requirements at the field and plot scales. The ground platform is mainly suitable for small-scale crop growth monitoring, which is affected by the scope of data acquisition and the cost of use, and cannot achieve rapid monitoring at spatial scales.

In recent years, the continuous development of UAV flight platforms and airborne sensor technology has promoted the application of UAV remote sensing technology in agricultural and forestry information monitoring. UAV remote sensing platforms have the advantages of low cost, simple structure, high mobility and high spatial and temporal resolution to make up for the shortage of satellite and ground-based remote sensing platforms (Xie and Yang, 2020). The UAV platform, equipped with visible, multispectral, and hyperspectral cameras, is used to acquire image data. Image processing techniques are applied to extract essential information, such as spectral features, texture, and point clouds, which are subsequently used to build models for crop growth parameter monitoring and yield estimation. (Liu et al., 2018; Sarkar et al., 2021; Jiang et al., 2022). The model construction process often involves a combination of non-linear relationships that affect the model’s universality. Machine learning methods can effectively solve the modelling problem of non-linear relational combinations and have been widely used in remote sensing monitoring. Wang and Jiang (2021) used UAV multispectral remote sensing data to achieve the monitoring of soybean leaf area index, and the support vector machine (SVM) had better predictions compared to the linear model (R^2^ = 0.688, RMSE = 0.016). Qi et al. (2020) achieved accurate estimation of peanut LAI (R^2^ = 0.968, RMSE = 0.165) using Back Propagation neural network algorithm (BPNN) combined with UAV spectral features. Kanning et al. (2018) extracted wheat canopy spectra based on UAV hyperspectral images and constructed a model for monitoring wheat LAI and chlorophyll using Partial Least Squares Regression (PLSR), demonstrating the feasibility of UAV hyperspectral imaging technology for monitoring crop growth parameters at the field scale. Although spectral features combined with machine learning algorithm can better estimate crop LAI, when LAI is high, the estimation model constructed by various vegetation indexes will appear “over-fitting” phenomenon (Hang et al., 2021). In addition, UAV multispectral images offer limited spectral information, and relying solely on spectral parameters such as reflectance or vegetation index may result in “same spectrum and different things” or “same thing and different spectrum” scenarios (Liu et al., 2018). Thus, crop growth monitoring should incorporate multiple data dimensions, such as time and space, to account for spectral, temporal, and spatial resolutions.

Texture features are also one of the image features of UAV remote sensing imagery (Yang et al., 2021), and are widely used for image classification and monitoring of crop growth physiological indicators (Haralick et al., 1973; Coburn and Roberts, 2004; Li et al., 2019). Chen et al. (2019) used spectral information and texture information to estimate chlorophyll content of potatoes and found that the fusion of vegetation index and texture features could significantly improve the estimation accuracy of the model. Zhu et al. (2022) developed a machine learning model for remote sensing monitoring of Wheat Scab using multispectral and texture features. Their results demonstrated that the fusion of vegetation indices and texture parameters improved the accuracy of Wheat Scab detection. However, little is known about field-scale remote sensing monitoring of maize LAI using spectral and texture features extracted from UAV multispectral imagery.

Based on the above problems, this study attempts to extract and optimize the spectral and texture features from UAV multispectral images. It combines the optimized features with SVM, Random Forest (RF), BPNN and PLSR to build a field-scale corn LAI remote sensing monitoring model. We compared the effects of spectral features and texture features on LAI estimation. Furthermore, we explored the influence of machine learning method synergistic spectral and texture features on LAI estimation potential.

## 2 Materials and methods

### 2.1 Experimental design

**Figure 1:**
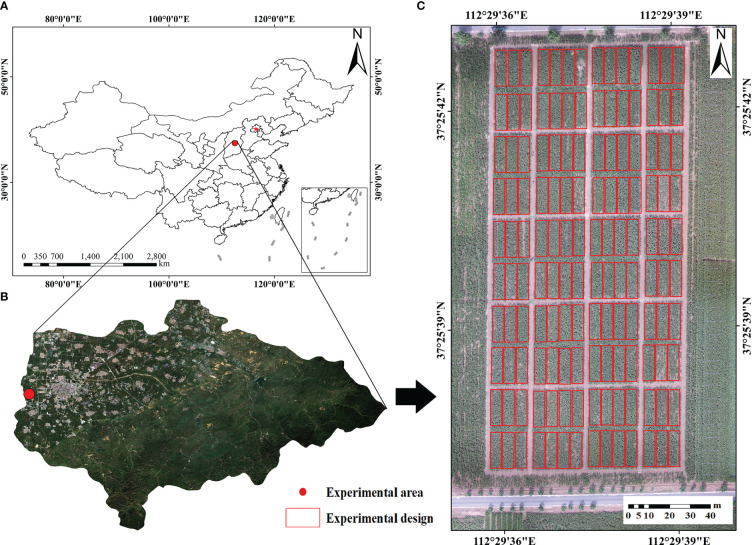
Overview of the study area. **(A)** Geographical location of Taigu District **(B)** the location of the experiential area **(C)** the design of the field experience.

This experiment was conducted in the maize experimental field of Shanxi Agricultural University, Jinzhong City, Shanxi Province (37°25′ N, 112°29′ E) (**Figure 1**).

The experimental area is located in Taigu District, with an average altitude of about 780 m, belonging to a temperate continental monsoon climate, with an average annual temperature of 6-10 °, an average annual rainfall of 410-450 mm and a frost-free period of 160 days. The climatic conditions such as light, heat and water are suitable for maize growth.

The experiment was conducted in a single-factor design with 140 maize varieties (Xinyu 303, Jinfeng 278, RP818, etc.), each planted on an area of 75 m^2^, total 140 plots. In five key growth periods of maize, namely, tasseling period (24 July, 2021), silking period (4 August, 2021), flowering period (14 August, 2021), filling period (25 August, 2021) and milk ripening period (8 September, 2021), LAI was measured in areas with high vegetation coverage and consistent growth.

### 2.2 UAV multispectral image acquisition

**Figure 2:**
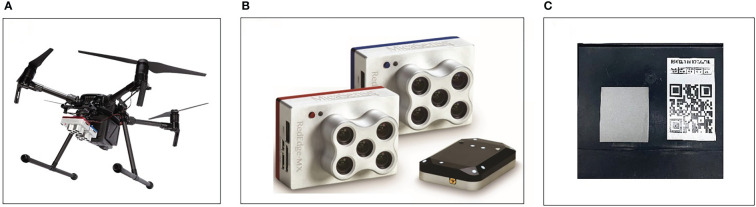
UAV near ground remote sensing platform. **(A)** Four rotors UAV **(B)** Multispectral imaging system **(C)** The calibration panel.

In this study, a Meridian M210 V2 quadcopter UAV (DJI Innovations, Shenzhen, China) (**Figure 2A**) with a RedEdge-MX imaging system (MicaSense, Seattle, WA, USA) (**Figure 2B**) was used as a high-throughput remote sensing platform to acquire multispectral images of maize during critical fertility periods. RedEdge-MX dual-camera imaging system has 10 spectral channels with a spectral range of 444-842 nm and can simultaneously obtain 10 discontinuous multispectral images with a resolution of 1280 × 960 pixels. The detailed band parameters are shown in **Table 1**. The flight time is between 10:00 and 12:00, the flight altitude is set to 60 m, and the forward overlap rate and side overlap rate are set to 85%.

**Table 1:** RedEdge-MX Dual multispectral cameras bands.

Band number	Band name	Center wavelength (nm)	Bandwidth (nm)
B1	Coastal Blue*	444	28
B2	Blue	475	32
B3	Green*	531	14
B4	Green	560	27
B5	Red*	650	16
B6	Red	668	14
B7	Red edge*	705	10
B8	Red edge	717	12
B9	Red edge*	740	18
B10	Near infrared	842	57

*Indicates the bands of the RedEdge-MX Blue camera.

### 2.3 UAV multispectral image processing

After the flight, the original multispectral images acquired by the UAV and the calibration plate (**Figure 2C**) images taken before takeoff were imported into Pix4D mapper (Pix4D S.A., Lausanne, Switzerland) together for image stitching and radiometric calibration. After the stitching was completed, the software automatically completed the radiometric calibration according to the DN value of the calibration gray plate and the reflectance calibration fitting equation. After completing the above processing process, the orthoimage of each waveband were obtained. The orthoimages were processed using ENVI Classic 5.5 (Harris Geospatial Solutions, Inc., Broomfield, CO, USA) for band fusion to obtain multispectral image data.

### 2.4 Field data collection

In this study, we selected the SunScan Canopy Analyser to measure maize LAI data while acquiring UAV multispectral images. During the data collection process, maize leaf area index was measured by selecting three inter-row shaded locations of 1 meter in length at random within each plot. The average of the three measurements was used as the LAI value for the plot.

### 2.5 Multispectral image features extraction

#### 2.5.1 Selection of vegetation index

By combining different bands linearly or nonlinearly, the vegetation indices constructed has certain indicative significance for the dynamic changes of vegetation canopy information, which not only reduces the influence of atmospheric and soil environmental factors but also enhances the sensitivity of LAI to canopy reflectivity. According to the previous research results (Qi et al., 2020; Hang et al., 2021), we selected eight commonly used vegetation indices to estimate LAI, and the specific calculation formulas are shown in **Table 2**.

**Table 2:** Formula for calculating vegetation indices.

Vegetation Index	Description Formula	Reference
Normalized Difference Vegetation Index (NDVI)	(NIR_842_-R_650_)/(NIR_842_+R_650_)	(Rouse et al., 1974)
Red Edge Normalized Difference Vegetation Index (NDRE)	(NIR_842_-RE_705_)/(NIR_842_+RE_705_)	(Gitelson et al., 1996)
Modified Triangular Vegetation Index (MTCI)	(NIR_842_-RE_705_)/(RE_705_+R_650_)	(Dash and Curran, 2004)
Difference Vegetation Index (DVI)	NIR_842_-R_650_	(Naito et al., 2017)
Ratio Vegetation Index (RVI)	NIR_842_/R_650_	(Liang et al., 2013)
Red edge chlorophyll index (CI_red edge_)	NIR_842_/RE_705_-1	(Gitelson et al., 1996)
Enhanced Vegetation Index (EVI)	2.5((NIR_842_-R_650_)/(NIR_842_+6R_650_-7.5B_475_+1))	(Prabhakara et al., 2015)
Soil-adjusted Vegetation Index (OSAVI)	(1+0.16)(NIR_842_-R_650_)/(NIR_842_+R_650_+0.16)	(Rondeaux et al., 1996)

B_475_, R_650_, RE_705_ and NIR_842_ in the table represent the reflectance at blue, red, red edge and near-infrared bands, respectively.

#### 2.5.2 Texture features extraction

To improve computational efficiency, we select three bands of red 650 nm, red edge 705 nm and near-infrared 842 nm for texture feature extraction. Grey level co-occurrence matrix (GLCM) is one of the most widely used methods in texture feature extraction (Haralick et al., 1973). This method was proposed by Haralick in 1973, and is mainly used in machine vision, image classification, image recognition and so on (Kavdir and Guyer, 2004; Adjed et al., 2018; Vani et al., 2018). After radiation correction and image fusion, eight texture features such as Mean (mean), variance (var), homogeneity (hom), contrast (con), dissimilarity (dis), entropy (ent), second moment (sm) and correlation (cor) were extracted using the GLCM. A total of 24 texture feature values were selected and the mean value extracted from the region of interest was used as the texture feature value for the corresponding image.

In order to fully explore the application potential of texture features in UAV multispectral images in maize LAI estimation, this study used Matlab 2020a software(MathWorks, Natick, Massachusetts, USA) to traverse and combine eight texture feature values of three-band images and calculates three texture indexes: normalized difference texture index(NDTI) (Zheng et al., 2019), ratio texture index(RTI) and difference texture index(DTI) (Hang et al., 2021). The specific calculation formulas are as follows:


(1) $${\text{NDTI=(T}}_{1}{\text{-T}}_{2}{\text{)/(T}}_{1}{\text{+T}}_{2})$$

(2) $${\text{RTI=T}}_{1}{\text{/T}}_{2}$$

(3) $${\text{DTI=T}}_{1}{\text{-T}}_{2}$$

In the formula, T_1_ and T_2_ are texture eigenvalues of random bands.

### 2.6 Model construction and evaluation

In this study, a total of 700 datasets were collected from five key fertility periods of maize, each containing ground-truthed LAIs and UAV image features such as vegetation indices and texture indices. To ensure that each dataset could be involved in modeling and validation, the datasets were randomly divided into 10 parts using ten-fold cross-validation, with 90% (630 datasets) used for modeling and 10% (70 datasets) for model validation. Each model was trained 10 times to ensure robustness.

We developed 12 LAI estimation models for multiple fertility stages by combining ground truth LAIs with various inputs using four machine learning algorithms, namely SVM, RF, BPNN, and PLSR. The model inputs consisted of univariate and multivariate factors, where the former comprised vegetation indices and texture indices, and the latter involved their combination.

(1) The support vector machine is a popular machine learning algorithm for pattern recognition and nonlinear regression(Cortes and Vapnik, 1995). In this study, we used the SVM algorithm with a radial basis function (RBF) to construct maize LAI estimation models using various predictors. The SVM model requires tuning of the penalty factor c and the kernel function parameter g. After continuous testing, we determined the optimal values of c=1.00 and g=3.03.
(2) Random forest is a nonlinear regression modeling method based on multiple decision trees. It consists of two methods, Bootstrap sampling and Random subspace(Breiman, 2001), and is effective in handling high-dimensional data and covariance problem among variables, with strong noise resistance. The number of decision trees (ntrees) and the number of predictors randomly selected for each split (mtry) are the main parameters that need to be tuned to optimize the performance of a random forest model. After repeated testing, this study set the random forest parameters to ntrees=200 and tuned mtry according to different input variable types.
(3) Back Propagation (BP) based neural network consists of three parts: input layer (input), hidden layer (hidden), and output layer (output). To ensure the model monitoring accuracy, the BPNN model was trained several times and the model parameters were iteratively tuned. Finally, the learning rate of 0.01, 10 hidden layers and 1 output layer were used as the best parameters.
(4) PLSR originates from the nonlinear iterative partial least squares (NUPALS) algorithm proposed by Herman Wold et al. (2001). The number of latent variables (LV) is one of the important influencing factors to determine the prediction accuracy of the PLSR model, and in this study the model automatically adjusted the number of latent variables according to the input variable types.

In this study, coefficient of determination (R^2^), root mean square error (RMSE) and relative percent error (RPD) were used as evaluation indexes to evaluate the model performance. When the estimated model has higher R^2^, RPD and smaller RMSE in modeling and validation datasets, it indicates that the model has higher goodness of fit, accuracy and stability.

## 3 Results

### 3.1 Correlation analysis between spectral parameters and LAI

**Figure 3:**
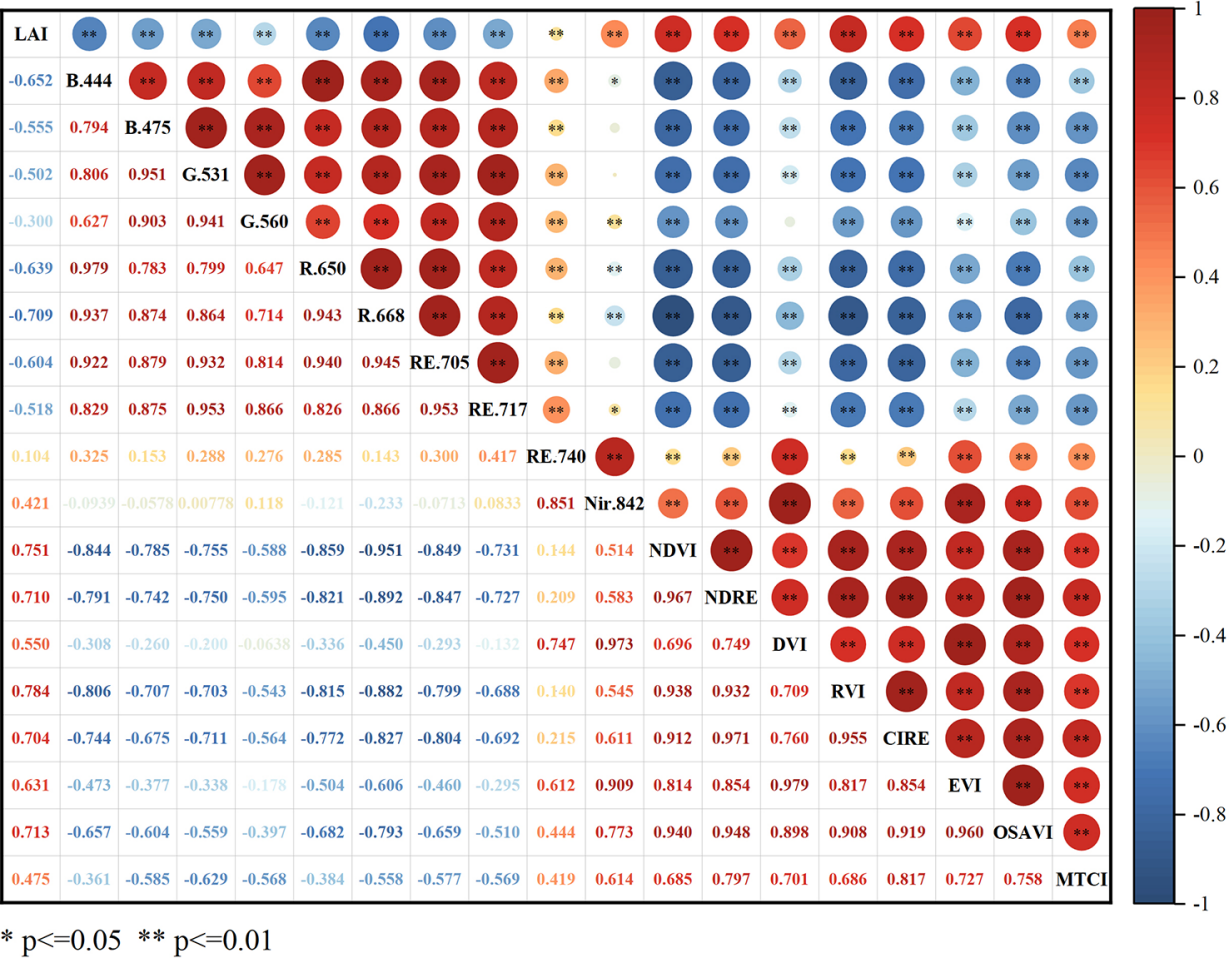
Correlation coefficient between specific parameters and maize LAI. * and * * are significant at 0.05 and 0.01 levels, respectively. The red area indicates positive correlation, the blue area indicates negative correlation. Darker colors and larger circles mean a stronger correlation between LAI and spectral parameters.

The correlation analysis of the 18 spectral parameters with LAI is shown in **Figure 3**. The correlation between LAI and canopy reflectance was significantly negative (p < 0.01) in the range of 444-717 nm and positive (p < 0.01) in the range of 740-842 nm. All eight selected vegetation indices were highly significantly positively correlated with LAI (P < 0.01), with RVI being the most strongly correlated with LAI (r = 0.784) and NDVI, OSAVI, NDRE and CI_red edge_ being more strongly correlated with LAI, with correlation coefficients above 0.700. All five vegetation indices included the three bands, red, red edge and NIR, indicating that the band combinations could be better for maize LAI monitoring.

### 3.2 Correlation analysis between LAI and texture features

The correlation analysis of the three bands of texture features with LAI (**Table 3**) showed that the mean texture values in the Red and NIR band were significantly correlated with LAI, with a strong correlation (r = -0.687 and -0.703).

**Table 3:** Correlation coefficients between texture features of the three bands and LAI.

Texture Features	Correlation Coefficients		
	Red_650_	Red edge_705_	NIR_842_
Mean(mean)	-0.687^**^	-0.703^**^	-0.095^*^
Variance (var)	-0.311^**^	-0.121^**^	0.055
Homogeneity (hom)	0.285^**^	0.073	-0.206^**^
Contrast (con)	-0.285^**^	-0.076^*^	0.084^*^
Dissimilarity (dis)	-0.291^**^	-0.071	0.131^**^
Entropy (ent)	-0.369^**^	-0.144^**^	0.188^**^
Second moment (sm)	0.357^**^	0.165^**^	-0.196^**^
Correlation (cor)	-0.088	0.036	0.237^*^

*and ** are significant at the 0.05 and 0.01 levels.

**Figure 4:**
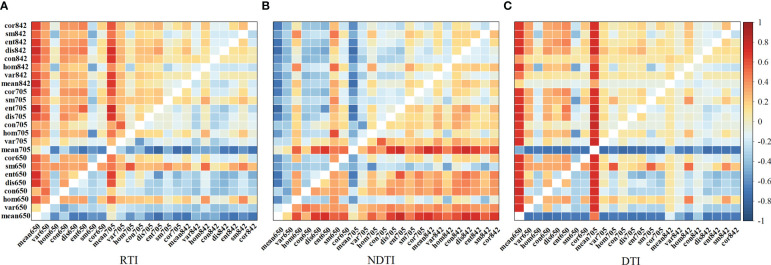
Correlation coefficient matrix between LAI and three types of texture index indices **(A)** the ratio texture index, **(B)** the normalized difference texture index, **(C)** the difference texture index. Each cell in the figure represents the correlation coefficient between the texture index, which is obtained by linearly transforming the original texture parameters corresponding to the x and y coordinates of each cell, and the LAI.

Due to the weak correlation between most texture features and LAI, this study constructed three texture indices composed of different texture feature values in order to improve the potential application of texture features in monitoring maize LAI. The results shown in **Figure 4** indicated that the correlation between the linearly transformed texture features and LAI was significantly enhanced. Among them, the RTI (mean_705_, ent_705_) had the strongest correlation with LAI, with a correlation coefficient of -0.804, which was a 14.370% increase in the absolute value of the correlation coefficient compared to the red edge mean.

### 3.3 Comparison of LAI estimation models based on vegetation indices and texture indices

In order to fully investigate the potential of combining UAV spectral and texture features with machine learning algorithms for LAI estimation. Based on the strength of the correlation between different image features and LAI, we selected five vegetation indices (RVI, NDVI, OSAVI, NDRE and CI_red edge_) and three texture indices (RTI (mean_705_, ent_705_), DTI (mean_705_, con_705_) and NDTI (mean_650_, ent_705_)) as independent variables, and constructed LAI estimation models by using four machine learning algorithms: SVM, RF, BPNN and PLSR respectively. **Table 4** shows the training results of machine learning models with different input variables. In the single variable model, from the perspective of modelling methods, RF performed best in the dataset of vegetation indices and SVM performed best in the dataset of texture indices; From the perspective of the different input variables, the estimation model based on the texture indices performs better overall than the vegetation indices when using the same modelling approach. On the whole, the estimation of the SVM model based on TIs was optimal (R^2^ = 0.790, RMSE = 0.312, RPD = 2.010).

**Table 4:** Summary of the results of estimating LAI by machine learning models based on different inputs.

Technique	Data set	VIs			TIs			VIs+TIs		
		R^2^	RMSE	RPD	R^2^	RMSE	RPD	R^2^	RMSE	RPD
SVM	Cal	0.698	0.371	1.487	0.735	0.348	1.656	0.806	0.315	1.856
	Val	0.621	0.433	1.338	0.790	0.312	2.010	**0.813**	**0.297**	**2.084**
BPNN	Cal	0.648	0.403	1.335	0.689	0.380	1.445	0.791	0.311	1.899
	Val	0.664	0.392	1.716	0.688	0.389	1.787	0.795	0.330	1.936
RF	Cal	0.840	0.271	2.257	0.844	0.269	2.095	0.906	0.208	3.149
	Val	0.733	0.396	1.545	0.699	0.411	1.399	0.786	0.347	1.888
PLSR	Cal	0.649	0.402	1.392	0.652	0.397	1.405	0.753	0.335	1.755
	Val	0.616	0.413	1.328	0.660	0.425	1.474	0.759	0.347	1.775

The value in bold type indicates that the model is the best LAI estimation model. The Cal and Val in the table represent the calibration set data set and validation set data set respectively.

Using vegetation and texture indices as multivariate input variables to construct the LAI estimation model. From the perspective of modelling method, the RF model in the calibration set performed the best from the perspective of the modelling approach (R^2^ = 0.906, RMSE = 0.208, RPD = 3.149), with the SVM model performing second best (R^2^ = 0.806, RMSE = 0.315, RPD = 1.856). However, in the validation set, the SVM model performed best (R^2^ = 0.813, RMSE = 0.297, RPD = 2.084). In contrast, the R^2^ of the RF model plummeted from 0.906 to 0.786, the RMSE increased by 66.827% and the RPD decreased by 40.044%. Although the monitoring effect of BPNN and PLSR is slightly weaker than that of SVM and RF, the estimation accuracy and model stability are also better (R^2^ > 0.75, RPD > 1.75). The above results show that the SVM model has the best estimation accuracy and stability, and the other three models also have great prediction results.

When analyzed from the perspective of the input variables, the machine learning models constructed by fusing the two types of indices explained significantly more variance in the LAI compared to the single-factor input variables of the vegetation or texture indices. Combining the VIs with the TIs resulted in R^2^ means of 0.817 and 0.788, RMSE means of 0.292 and 0.330, and RPD means of 2.165 and 1.921 for the calibration and validation sets, respectively. Compared with the single vegetation indices data source model, R^2^ increased by 14.810% and 19.757%, RMSE decreased by 19.337% and 19.118%, and RPD increased by 33.807% and 29.622%. Compared with the single texture indices model, R^2^ increases by 11.507% and 11.142%, RMSE decreases by 16.092% and 14.063%, and RPD increases by 31.212% and 15.237%. The above results show that the estimation effect of the model is obviously improved and more stable after fusing different image features.

**Figure 5:**
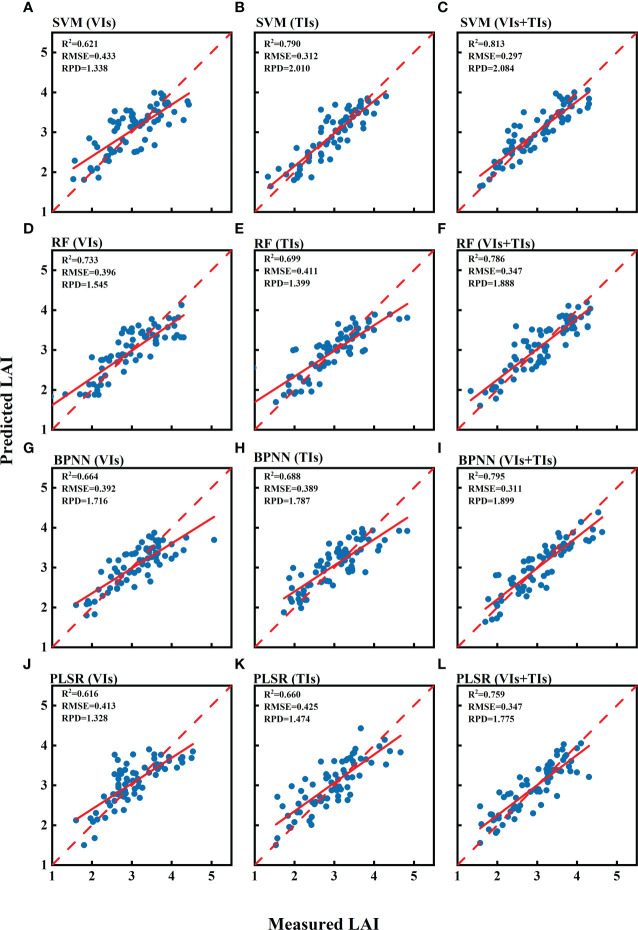
Accuracy evaluation results of LAI estimation models based on vegetation indices (VIs), texture indices (TIs) and combined vegetation indices and texture indices (VIs+TIs) in the validation set. The models evaluated are SVM, RF, BPNN, and PLSR, shown respectively in **(A–C)**, **(D–F)**, **(G–I)**, and **(J–L)**.

The scatter plot in **Figure 5** showed good consistency between the predicted LAI values from the machine learning estimation model and the measured LAI values in the validation dataset, with an RMSE ranging from 0.297 to 0.433 and an RPD ranging from 1.328 to 2.084. Combining vegetation indices and texture indices resulted in the best estimation results among the four types of machine learning models.

## 4 Discussion

UAV remote sensing has great potential in the process of crop phenotype information mining and analysis due to its high spatial and temporal resolution and simple operation (Xie and Yang, 2020). In this study, multispectral images of the study area were acquired by using a UAV with a multispectral camera. The non-destructive and rapid estimation of maize LAI at the plot scale was achieved by extracting different types of image features and combining them with machine learning algorithms.

### 4.1 Analysis of monitoring LAI by vegetation indices

Vegetation indices are widely used in crop chlorophyll content (Jiang et al., 2022), LAI (Li et al., 2019), biomass (Gnyp et al., 2014) and yield prediction (Fu et al., 2020; García-Martínez et al., 2020). Crop canopy reflectance is easily influenced by leaf pigmentation in the visible bands, which can lead to “oversaturation” of the vegetation indices (Hatfield et al., 2008). In contrast, red-edge and near-infrared reflectance are mainly influenced by canopy structure and have a stronger penetration effect. Hence, researchers usually choose red-edge and near-infrared bands to construct vegetation indices. Shi et al. (2022) demonstrated that vegetation indices based on red light bands and near-infrared bands correlate well with LAI and AGB of red and green beans, allowing for growth monitoring of intercropped crops in plot tea plantations. Zheng et al. (2019) found that the vegetation indices based on red-edge bands were important parameters for rice biomass estimation before tasseling. However, the estimation was significantly reduced after tasseling, mainly because canopy leaf biomass was sensitive to red-edge bands but not stems. Qi et al. (2020) used fixed-wing UAV to monitor peanut growth, and found that red light and near-infrared bands were sensitive bands of peanut LAI, which can effectively predict the changes of peanut LAI. In this study,we found strong correlations (r > 0.700) between maize LAI and five vegetation indices: RVI, NDVI, OSAVI, NDRE, and CI_red edge_. These indices were identified as effective for monitoring LAI in various maize varieties. The results are in general agreement with the results of previous studies, indicating that spectral indices based on red light, red edge and near-infrared bands are of good application in crop monitoring and can achieve rapid and non-destructive monitoring of crop growth parameters.

In this study, DVI and MTCI performed poorly in estimating LAI, and the correlation coefficients were only 0.550 and 0.475. There may be two possible reasons for this result: (1) the influence of other disturbing factors such as soil background and vegetation shading on the multispectral reflectance; (2) the high LAI level in the middle and late stages of maize growth, resulting in an underestimation of some vegetation indices. In the process of monitoring crop growth using UAV multispectral imagery, the use of spectral features alone may not achieve satisfactory results (Zheng et al., 2019; Yang et al., 2021; Fei et al., 2022). The use of vegetation indices alone can only quantitatively analyze the structural characteristics, biochemical components and productivity trends of crop canopies from a spectral perspective. It cannot deeply explore the effective information of other data dimensions in UAV multispectral imagery.

### 4.2 Estimation potential analysis of texture features

Texture features can reveal changes in crop canopy information from the data dimension of image spatial features. Previous work has used texture parameters from satellite remote sensing data combined with spectral and topographic features to estimation above-ground biomass in forests, demonstrating the feasibility of applying texture parameters in agricultural remote sensing (Lu and Batistella, 2005; Li et al., 2008). In this study, most of the texture parameters had poor correlation with LAI. However, the correlation between the constructed texture indices and LAI were significantly improved after linear processing. The texture indices RTI (mean_705_, ent_705_), DTI (mean_705_, con_705_) and NDTI (mean_650_, ent_705_) effectively improved the estimation of LAI. Similar conclusions were obtained by Hang et al. (2021) and Fei et al. (2022) when using texture parameters for rice growth monitoring and yield estimation of wheat. This is mainly because the texture is linearly combined and transformed to reduce the influence of soil background, vegetation shading and topographic factors, which can better highlight the changing patterns of feature characteristics.

### 4.3 Influence of UAV image features fusion on LAI estimation potential

The multi-input model based on vegetation and texture indices in this study was superior to the model with a single input variable, with significant improvements in fit, estimation accuracy and stability. In particular, the model combining UAV texture and spectral features outperformed the model using only the vegetation indices, with a 19.757% increase in R^2^, a 19.118% decrease in RMSE and a 29.622% increase in RPD in the validation set. The results of this study are similar to those of previous studies. Ma et al. (2022) used color indices and texture features from UAV RGB images to accurately estimate cotton yield, with the RF_ELM model based on color indices and texture features having the highest accuracy (R^2^ = 0.911). Yang et al. (2021) used vegetation indices and texture features to achieve an estimation of LAI for rice at full fertility. The combination of spectral features and texture features had superior predictive power than vegetation indices. In summary, combining the UAV spectral features with texture features is an effective method to improve the accuracy of LAI estimation.

### 4.4 Comparison of different machine learning models

Machine learning algorithms combined with remote sensing data have been widely used in areas such as crop growth monitoring(Li et al., 2019; Zheng et al., 2019; Zhang et al., 2021), yield estimation (Fu et al., 2020; García-Martínez et al., 2020; Ma et al., 2022) and disease identification (Guo et al., 2021; Zhu et al., 2022). This study used four machine learning algorithms, SVM, RF, BPNN and PLSR, to construct LAI monitoring models for different maize varieties. The results show that the SVM model performs best as a whole, and the model’s training results and verification results have a high degree of explanation for the variation of LAI. In the machine learning models constructed based on vegetation and texture indices, the validation sets R^2^ and RPD of SVM were improved and RMSE decreased compared to RF, PLSR and BPNN models, indicating the high performance of SVM modelling. Zhu et al. (2022) realized high-precision monitoring of wheat scab by using machine learning method combined with spectral and texture features of drones, and the model built by SVM in collaboration with VIs + TFs can provide the most accurate monitoring results; Omer et al. (2016) used WorldView-2 multispectral imagery combined with SVM and ANN algorithms to achieve monitoring of LAI of forest endangered tree species, where the SVM model showed excellent prediction accuracy and model stability. The excellent performance of SVM model in LAI estimation may be related to the model structure. SVM uses the principle of structure minimization (Camps-Valls et al., 2006) to solve the nonlinear mapping problem between input variables and response variables. However, to address the issue of unstable RF model performance, a similar situation has been found in other studies, Han et al. (2019) used the cooperative machine learning algorithm of canopy structure information and spectral information to construct maize AGB, the performance of RF model in the training set and test set was quite different. There are two main reasons for the instability of the RF model: (1) the amount of data in the validation set is far less than that in the calibration set, and RF will perform better in extensive sample data; (2) the existence of outliers in the validation set due to human measurement problems reduces the stability of the model.

In this paper, when using different machine learning algorithms to estimate LAI with multivariate input variables, all four machine learning models achieved good performance, indicating that there is a non-linear relationship between the response variables and the various predictors. However, the input variables were selected without deeper mining of the input variable feature selection, and the contribution of different predictors to the LAI estimation model was not considered. To improve the monitoring accuracy of LAI, it is necessary to study the above shortcomings in future research.

## 5 Conclusions

Rapid and non-destructive plot-scale maize LAI estimation is important for UAV remote sensing monitoring of crop growth as well as precise agricultural management. In this study, we used image analysis techniques to extract spectral and texture features from UAV multispectral images and used machine learning methods (SVM, RF, BPNN, PLSR) to achieve fast and accurate estimation of maize LAI. Most Vegetation indices based on red, red-edge, and NIR bands exhibited strong correlation with LAI, whereas most texture features demonstrated limited association with LAI. Nevertheless, after applying linear transformation, texture indices displayed a substantially enhanced correlation with LAI. Among the different types of estimation models, the model constructed by SVM method combined with vegetation indices and texture indices was the best for LAI estimation (R^2 =^ 0.813, RMSE=0.297, RPD=2.084), and this result revealed that there was a non-linear relationship between LAI and spectral parameters and texture parameters. The results of this study show that the use of UAV near-ground remote sensing combined with image analysis techniques can achieve accurate monitoring of the growth of different maize varieties and provide guidance for maize variety selection.

## Data availability statement

The raw data supporting the conclusions of this article will be made available by the authors, without undue reservation.

## Author contributions

Conceptualization, XinS, WY and MF. data curation, XinS, ZY, PS and ZW. formal analysis, XinS and CY. funding acquisition, WY and MF. investigation, XinS, ZY, PS, KW and ZW. project administration, WY and MF. resources, WY and MF. writing—original draft, XinS. writing—review and editing, WY and MF. All authors contributed to the article and approved the submitted version.
